# Genetic and molecular pathways controlling rice inflorescence architecture

**DOI:** 10.3389/fpls.2022.1010138

**Published:** 2022-09-28

**Authors:** Yan Chun, Ashmit Kumar, Xueyong Li

**Affiliations:** ^1^ National Key Facility for Crop Gene Resources and Genetic Improvement, Institute of Crop Sciences, Chinese Academy of Agricultural Sciences, Beijing, China; ^2^ College of Agriculture, Fisheries and Forestry, Fiji National University, Nausori, Fiji

**Keywords:** rice (*Oryza sativa* L.), inflorescence, meristem, identity conversion, panicle branch, phytohormone

## Abstract

Rice inflorescence is one of the major organs in determining grain yield. The genetic and molecular regulation on rice inflorescence architecture has been well investigated over the past years. In the present review, we described genes regulating rice inflorescence architecture based on their roles in meristem activity maintenance, meristem identity conversion and branch elongation. We also introduced the emerging regulatory pathways of phytohormones involved in rice inflorescence development. These studies show the intricacies and challenges of manipulating inflorescence architecture for rice yield improvement.

## Introduction

**Figure 1:**
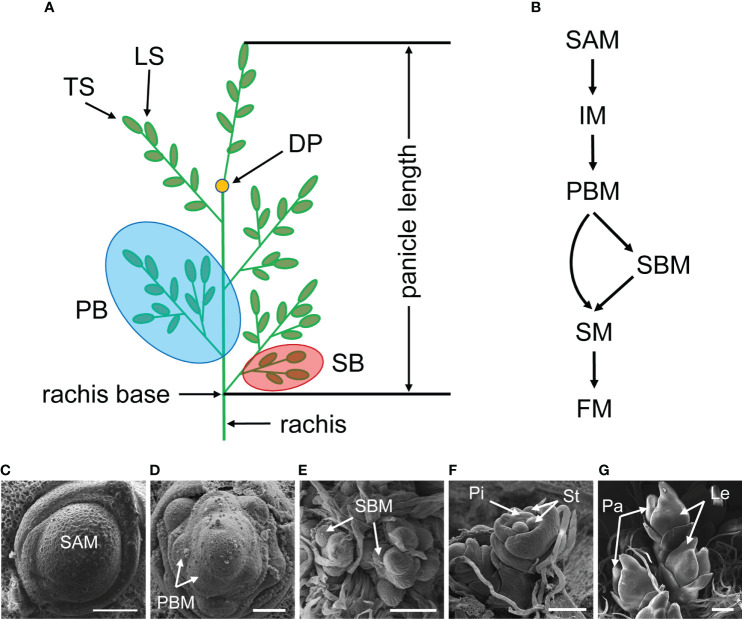
Rice inflorescence architecture and development process. **(A)** Schematic diagram of the rice inflorescence. The rice inflorescence is also called “panicle” that primarily contains a rachis, branches and spikelets. DP, degenerate point of rachis; PB, primary branch; SB, secondary branch; LS, lateral spikelet; TS, terminal spikelet. **(B)** A hypothesis of meristem identity conversion. SAM, shoot apical meristem; IM, inflorescence meristem; PBM, primary branch meristem; SBM, secondary branch meristem; SM, spikelet meristem; FM, floral meristem. **(C-G)** Scanning electron micrographs (SEM) showing early stages of rice inflorescence development. Pi, pistil; St, stamen; Pa, palea; Le, lemma. Bars = 50 μm in **(C)** and **(D)**; Bars = 100 μm in **(E, F)**; Bar = 200 μm in **(G)**.

Rice (*Oryza sativa* L.) is one of the major crops in the world, providing energy for over half of the population on earth (Sreenivasulu et al., 2021). Rice grain yield is influenced by a variety of factors, one of which is the rice inflorescence, also known as the panicle. A rice inflorescence is typically composed of a main rachis, primary branches (PB), secondary branches (SB), and spikelets. The spikelets are divided into two types: lateral spikelets (LS) and terminal spikelets (TS) which grow on the tips of branches (**Figure 1A**). Based on the quantity and length of these organs, the inflorescence architecture can be categorized into different types, such as short or long, erect or dense.

Rice inflorescence, like most other aboveground organs, is generated from a group of cells referred to as the shoot apical meristem (SAM). SAM initiates in the embryo and continues to generate leaves throughout the vegetative stage by maintaining its activity. During the reproductive stage, SAM converts to inflorescence meristem (IM) and produces two types of lateral meristem: primary branch meristem (PBM) and secondary branch meristem (SBM). Both meristems are undetermined until they acquire the terminated identity, at which point branch meristem (BM) differentiates into spikelet meristem (SM). Subsequently, floral organs, such as pistil, stamen, lemma and palea are developed, eventually forming a complete spikelet (**Figures 1B–G**). The branches begin to elongate during the differentiation stage of SM, which is another event crucial for inflorescence morphology.

In the past years, our understanding of genetic and molecular mechanisms that underpin rice inflorescence architecture has advanced significantly. These studies support breeders to create ideal inflorescence architecture of rice. Here, we summarize current advancements in elucidating the mechanisms of rice inflorescence development, with an emphasis on meristem activity maintenance, meristem identity conversion, branch elongation, and phytohormone regulation. We also explore the flaws of present researches and the challenges for future studies.

## Maintenance of SAM activity

**Figure 2:**
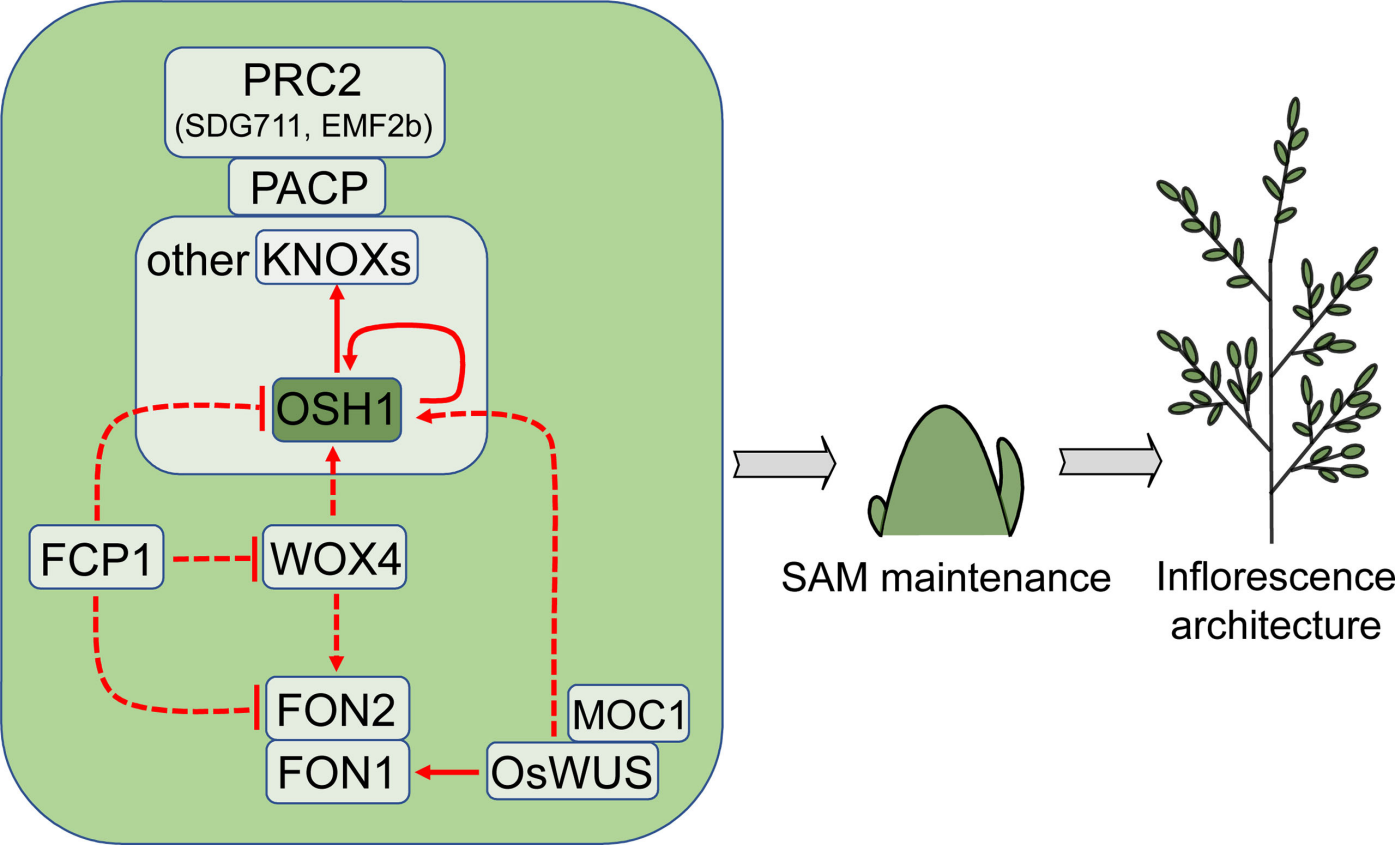
Regulators that control SAM or IM activity and affect inflorescence architecture. *OSH1* is a critical factor that is commonly used as a marker gene for rice SAM maintenance. Arrows and blocked arrows represent positive and negative regulation, respectively. Solid and dashed lines represent direct and indirect regulation, respectively. Red lines represent regulation at transcription level. SAM, shoot apical meristem; IM, inflorescence meristem.

SAM is formed during embryogenesis and keeps itself from differentiating through continuous cell division and vegetative development (Laux and Schoof, 1997). Rice IM directly develops from SAM, thus SAM activity has a significant impact on inflorescence architecture and yield. Despite several genes have been identified (**Figure 2**), the mechanism of SAM establishment and maintenance in rice is not as clear as that in Arabidopsis (Guan and Jiao, 2020; Wang et al., 2020; Eshed, 2021; Li et al., 2022).

Rice SAM establishment and maintenance are dependent on CLASS 1 KNOTTED1-LIKE HOMEOBOX (KNOX) family, among which *ORYZA SATIVA HOMEOBOX1* (*OSH1*) is expressed throughout SAM but not in leaf primordia (Sentoku et al., 1999). The expression of *OSH1* precedes organ differentiation and continues until floral organs formation (Sato et al., 1996). *OSH1* deficiency results in undersized rice inflorescence and fewer spikelets. As a transcription factor, OSH1 directly regulates itself and other *KNOX* genes (Tsuda et al., 2011).

OSH1 and several other KNOX proteins physically interact with PRC2-associated coiled-coil protein (PACP) in SAM (Tan et al., 2022). PACP is involved in the maintenance of H3K27me3 and inhibition of cell differentiation-promoting genes targeted by KNOXs in SAM through recruiting PRC2 complex proteins, including SDG711 and EMF2b. The growth of the *pacp* mutant is severely repressed due to the underdevelopment of SAM, leading to a much smaller inflorescence compared with wild type (Tan et al., 2022). These phenotypes are similar to those of the *SDG711* and *EMF2b* RNAi plants (Liu et al., 2015b).

In Arabidopsis, the negative feedback regulatory module of *WUSCHEL*-*CLAVATA* (*WUS*-*CLV*) mediates the maintenance of stem cells in SAM (Mayer et al., 1998; Schoof et al., 2000; Aichinger et al., 2012; Somssich et al., 2016). Interestingly, *OsWUS* in rice is involved in the formation of axillary meristems rather than SAM, suggesting that the function of these two genes may be distinct (Lu et al., 2015b; Tanaka et al., 2015). One explanation for this difference is that *OsWUS* is specifically expressed in lateral precursor meristem instead of SAM (Tanaka et al., 2015). *OSH1* expression is down-regulated in the loss-of-function mutant of *OsWUS*, suggesting that *OsWUS* maintains axillary meristem activity by promoting *OSH1* expression (Tanaka et al., 2015). The Arabidopsis WUS directly interacts with STM, the ortholog of OSH1, coordinately maintaining SAM activity (Su et al., 2020), whereas it is unknown whether this mechanism is conserved in rice.

*FLORAL ORGAN NUMBER1* (*FON1*) and *FON2* (also known as *FON4*) in rice are the homologs of *CLV1* and *CLV3*, respectively. The loss-of-function mutants *fon2*/*fon4* and *fon1* produce inflorescences with more primary branches due to the increased size of SAM or IM (Suzaki et al., 2004; Chu et al., 2006; Moon et al., 2006; Suzaki et al., 2006). *CLV1* is mainly expressed in the central zone of SAM in Arabidopsis (Clark et al., 1997), but *FON1* is expressed throughout the SAM and BM in rice (Suzaki et al., 2004). These studies show that the maintenance of meristem activity mediated by *CLV* signaling is conserved in rice, but the precise regulation may differ from that in Arabidopsis.

Current studies show that OsWUS can directly bind and enhance *FON1* expression, and this binding is reliant on another meristem activity regulator, MONOCULM1 (MOC1), which has been identified as a QTL controlling rice inflorescence architecture (Shao et al., 2019; Zhang et al., 2020). MOC1 physically interacts with and activates OsWUS to modulate *FON1* expression (Shao et al., 2019). Although *MOC1* is expressed in axillary meristem rather than SAM, its loss-of-function mutant showed reduced number of panicle branches and spikelets (Li et al., 2003; Zhang et al., 2020).

Rice SAM maintenance is also dependent on another plant-specific homeobox transcription factor, WUSCHEL-RELATED HOMEOBOX4 (WOX4), which belongs to the same WOX subfamily as OsWUS (Ohmori et al., 2013). Unlike *OsWUS*, the expression of *WOX4* is distributed throughout the SAM. At the reproductive stage, *WOX4* is expressed in BM and SM. Interference of *WOX4* expression results in a smaller SAM and down-regulation of *OSH1* and *FON2* (Ohmori et al., 2013). FON2-LIKE CLE PROTEIN1 (FCP1), a FON2-related CLE domain-containing protein, inhibits *WOX4* expression. Overexpression of *FCP1* prevents SAM formation and decreases the expression of *OSH1*, *FON2*, and *WOX4*, showing that *FCP1* plays a negative regulatory role in SAM maintenance (Ohmori et al., 2013).

## Conversion of meristem identity

**Figure 3:**
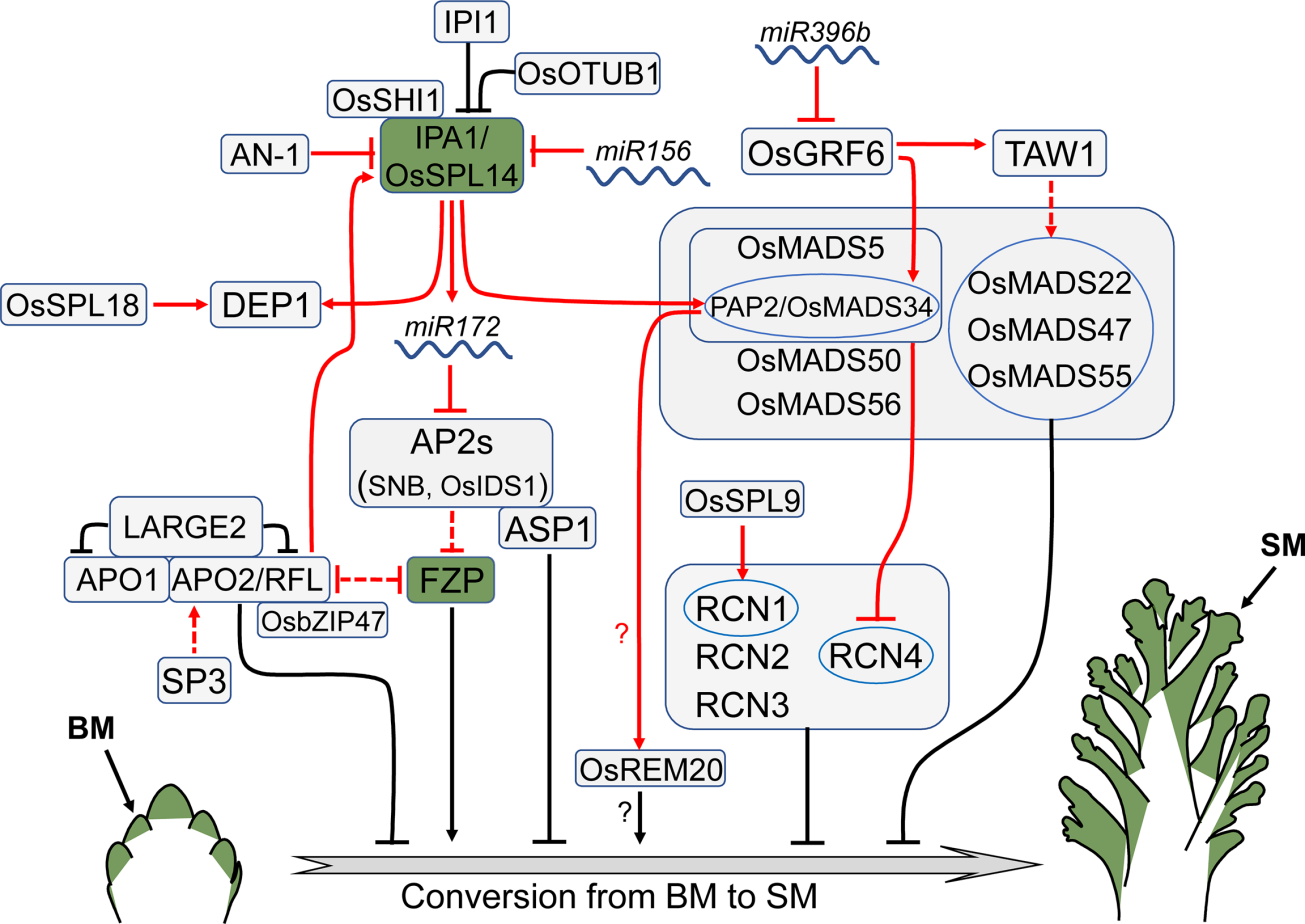
Regulators that control meristem identity conversion. *FZP* promotes the conversion of BM to SM, and its expression indicates that the meristem has acquired terminal identity. *IPA1*, also known as *WFP*/*OsSPL14*, is an important rice yield regulator that affects both meristem activity and identity conversion. Arrows and blocked arrows represent positive and negative regulation, respectively. Solid and dashed lines represent direct and indirect regulation, respectively. Red and black lines represent regulation between factors at transcriptional and protein level, respectively. BM, branch meristem; SM, spikelet meristem.

The conversion of SAM to IM and BM to SM are two main events in the establishment of rice inflorescence architecture. Delayed conversion may result in a larger inflorescence with more branches and spikelets. Many genes associated with meristem identity conversion have been identified *via* studies using Arabidopsis as a model, including *LEAFY* (*LFY*), *APETALA1* (*AP1*), and *CAULIFLOWER* (*CAL*). These genes enable the lateral meristem to acquire a floral identity and differentiate into flower organs (Mandel et al., 1992; Weigel et al., 1992; Kempin et al., 1995). In recent years, quite a few genes involved in the meristem identity conversion have also been identified in rice (**Figure 3**).

*FRIZZY PANICLE* (*FZP*), encoding an ERF (ethylene response factor) family transcription factor, promotes the conversion of BM identity to SM identity. The expression of *FZP* is restricted in a half-ring domain of SM before and during the formation of rudimentary glume meristem (Komatsu et al., 2003). In the loss-of-function *fzp* mutant, the generation of spikelets is prevented, leading to the formation of higher order branches instead of spikelets (Komatsu et al., 2003). On the contrary, overexpression of *FZP* severely inhibits the development of secondary branches, resulting in significantly reduced length of inflorescence and number of secondary branches and spikelets (Bai et al., 2016).

*FZP* is a major negative regulator of *ABERRANT PANICLE ORGANIZATION2* (*APO2*)/*RICE FLORICAULA LEAFY* (*RFL*), the rice ortholog of Arabidopsis *LFY* encoding a plant-specific transcription factor. The precise expression pattern of *APO2*/*RFL* in the vegetative stage remains controversial, since two studies on whether *APO2* is expressed in SAM reported opposite results (Kyozuka et al., 1998; Ikeda-Kawakatsu et al., 2012). In the reproductive stage, *APO2*/*RFL* is expressed in both PBM and SBM, but its expression is downregulated after the branch formation (Kyozuka et al., 1998; Ikeda-Kawakatsu et al., 2012). Expression of *APO2*/*RFL* is increased in young panicles of the *fzp* mutant, but reduced in the *FZP* overexpression plants (Bai et al., 2016). Similar to the *FZP* overexpression plants, the *apo2* loss-of-function mutant or transgenic lines with reduced *APO2*/*RFL* expression generate small inflorescence with decreased primary branches, due to the premature termination of BM (Rao et al., 2008; Ikeda-Kawakatsu et al., 2012). Interestingly, *FZP* is up-regulated in the *RFL* knock-down plants (Rao et al., 2008), implying mutual repression between *FZP* and *APO2*/*RFL*. The transcription factor OsbZIP47 interacts with APO2/RFL, suppressing BM identity conversion to SM. Knocking down *OsbZIP47* leads to a reduction in inflorescence axis length, primary branch number and spikelet number (Prakash et al., 2022).

*SHORT PANICLE3* (*SP3*), which encodes a Dof transcription factor, also regulates *APO2/RFL*. The expression of *APO2*/*RFL* is down-regulated in the young panicle of the *sp3* mutant, resulting in a reduction in panicle length, secondary branch number, and spikelet number (Huang et al., 2019). Another IM identity regulator APO1 directly interacts with APO2, coordinately controlling rice IM identity (Ikeda-Kawakatsu et al., 2012). APO1 is an F-box protein that positively controls panicle branch number and spikelet number by inhibiting the conversion of IM to SM. The expression of *APO1* is detectable in SAM and BM, particularly in the outer layers of the rachis meristem and PBM (Ikeda et al., 2007). The *apo1* mutant has fewer panicle branches and spikelets, whereas *APO1* overexpression results in opposite effects (Ikeda et al., 2007). Recent studies reveal that APO1 and APO2 physically interact with LARGE2, a HECT-domain E3 ubiquitin ligase OsUPL2. The APO1-APO2 complex is accumulated in the *large2* mutant, contributing to a larger inflorescence and increased grain number in comparison with wild type (Huang et al., 2021).

*ABERRANT SPIKELET AND PANICLE1* (*ASP1*) encodes a TOPLESS-related transcription corepressor, the rice homolog of Arabidopsis *TOPLESS* (*TPL*) which determines SAM fate (Long et al., 2006). Similarly, *ASP1* in rice is also involved in determination of meristem identity. The expression of *ASP1* is strong during the initiation of IM and BM. Similar to *APO1*, *ASP1* exhibits higher expression in the outer region than the inner region of BM (Yoshida et al., 2012). The *asp1* mutant shows shortening of primary branch and the reduction of spikelet number, as the result of early conversion of BM into SM (Yoshida et al., 2012). ASP1 interacts with several APETALA2 (AP2) family transcription factors, such as SUPERNUMERARY BRACT (SNB) and ORYZA SATIVA INDETERMINATE SPIKELET1 (OsIDS1), which positively regulate panicle branch number through inhibiting the acquisition of SM identity (Wang et al., 2015). The expression of *FZP* in the *snb osids1* double mutant precedes that in wild type, indicating that the BM of the *snb osids1* double mutant is transformed into SM in advance, contributing to fewer branches and spikelets (Lee and An, 2012). As the targets of *miR172*, *SNB* and *OsIDS1* are down-regulated in the *miR172* overexpression plants, which display a phenotype identical to the *snb osids1* double mutant (Lee and An, 2012).

*IDEAL PLANT ARCHITECTURE1* (*IPA1*), also known as *WEALTHY FARMER’S PANICLE* (*WFP*), simultaneously regulates tiller number and inflorescence size, shaping rice plant with ideal architecture (Jiao et al., 2010; Miura et al., 2010). *IPA1*, encoding a plant-specific transcription factor SQUAMOSA PROMOTER BINDING PROTEIN-LIKE 14 (OsSPL14), is a target of *miR156*. *IPA1* is expressed in both SAM and IM, especially highly in PBM and SBM (Jiao et al., 2010; Miura et al., 2010). Appropriately elevating *IPA1* expression, such as by interfering with *miR156* expression, altering the *miR156* target site in *IPA1* or reducing epigenetic repression of *IPA1*, can produce larger inflorescences and control the number of tillers at an optimal level, resulting in enhanced yield (Jiao et al., 2010; Miura et al., 2010; Wang et al., 2015; Zhang et al., 2017). However, overexpression of *IPA1* at high levels results in a small inflorescence with fewer branches and spikelets, particularly the secondary branches (Wang et al., 2015; Du et al., 2017). These morphological alterations may be achieved by regulating meristem identity conversion, as evidenced by the ectopic expression of *FZP* in BM of the *IPA1* overexpression plants (Wang et al., 2015; Du et al., 2017). *IPA1* is a directly target of APO2 in regulating inflorescence architecture. Overexpression of *IPA1* recovers the panicle defects of the *apo2* mutant, indicating that *APO2* acts upstream of *IPA1* (Miao et al., 2022).

Being a transcription factor, IPA1 binds directly to the promoter of *miR172* and activates its expression, thus inhibiting the *AP2* genes (Wang et al., 2015). IPA1 also directly targets and activates *DENSE AND ERECT PANICLE 1* (*DEP1*) (Lu et al., 2013). Another plant-specific transcription factor, ORYZA SATIVA SHORT INTERNODES1 (OsSHI1), is involved in this regulation (Duan et al., 2019a). OsSHI1 interacts with IPA1 and inhibits its transcriptional activity by affecting the binding of IPA1 to the *DEP1* promoter (Duan et al., 2019a). The level of IPA1 protein is regulated by the RING-finger ubiquitin E3 ligase, IPA1 INTERACTING PROTEIN1 (IPI1). IPI1 stimulates IPA1 degradation by adding K48-linked polyubiquitin chains in panicles but stabilizes IPA1 by adding K63-linked polyubiquitin chains in SAM. As a result, IPI1 controls panicle branching and tillering by differentially regulating IPA1 levels in various rice tissues (Wang et al., 2017a). IPA1 also interacts with the deubiquitinating enzyme OsOTUB1, which restricts the K63-linked ubiquitination of IPA1, in turn promoting IPA1 degradation through the K48Ub dependent-proteasome pathway. OsOTUB1 deficiency or reduced expression leads to the accumulation of IPA1, resulting in a large inflorescence (Wang et al., 2017b).

In Arabidopsis, several MADS-box genes determine floral meristem identity by directly inhibiting IM transition factor *TERMINAL FLOWER1* (*TFL1*) (Liu et al., 2013). This genetic mechanism seems conserved in rice. Loss-of-function mutation of *PANICLE PHYTOMER2* (*PAP2*)/*OsMADS34* prevents newly generated meristems from developing into SM, resulting in more branches and spikelets. However, a large number of branches and spikelets are aborted at basal nodes of the panicle (Gao et al., 2010; Kobayashi et al., 2010; Zhu et al., 2022). Inactivation of other MADS-box genes, such as *OsMADS5*, *SHORT VEGETATIVE PHASE* (*SVP*) (*OsMADS22*, *OsMADS47* and *OsMADS55*), *SUPPRESSOR OF OVEREXPRESSION OF CONSTANS 1* (*SOC1*) (*OsMADS50* and *OsMADS56*) in the *pap2*/*osmads34* mutant gives rise to further increase of inflorescence branches, even generates tertiary branches, but the numerous branches are severely aborted (Liu et al., 2013; Zhu et al., 2022). This phenotype resembles the rice plants overexpressing *RICE CENTRORADIALISs* (*RCNs*) which are the rice orthologs of Arabidopsis *TFL1* (Nakagawa et al., 2002; Zhu et al., 2022). Particularly, *RCN4* is highly and ectopically expressed in the PBM of *OsMADSs* multiple knock-down plants, demonstrating that these MADS-box genes promote inflorescence meristem identity conversion by suppressing *RCN4* expression (Liu et al., 2013). A recent study further confirms that OsMADS5 and OsMADS34 directly target *RCN4* (Zhu et al., 2022). However, overexpression of two *SVP* genes, *OsMADS22* and *OsMADS55* separately, delays conversion of BM to SM, leading to increased secondary branch number (Yoshida et al., 2013). These studies suggest that *SVP* genes and other MADS-box genes may play opposite roles in determining rice inflorescence architecture, and *SVPs* could directly suppress meristem identity conversion instead of promoting conversion through suppressing *RCNs* expression.

The *MADS*-*TFL1* module in controlling meristem identity conversion and inflorescence architecture is regulated by *SPL* genes, such as *SPL3* and *SPL9*, in Arabidopsis (Wang et al., 2009; Yamaguchi et al., 2009). This mechanism seems also conserved in rice, evidenced by that IPA1/OsSPL14 directly binds the *PAP2*/*OsMADS34* promoter and up-regulates its expression (Wang et al., 2015). Meanwhile, overexpression of *RCN1* in the *OsSPL14* knock-down plants can rescue the secondary branch defects, suggesting that *OsSPL14* acts upstream of *RCN1* in controlling conversion of BM to SM (Wang et al., 2015). Recently, *OsSPL9* has been identified as a positive regulator of secondary branch number, which directly activates *RCN1* expression (Hu et al., 2021a), implying that *SPLs* mediate meristem identity conversion may not always through MADS-box genes.

*REPRODUCTIVE MERISTEM20* (*OsREM20*), encoding a B3 domain transcription factor, is expressed in the reproductive meristems, including PBM, SBM and SM. The loss-of-function mutation of *OsREM20* generates small inflorescence with reduced grain number. OsMADS34 directly binds the CArG box-containing inverted repeat sequence in the *OsREM20* promoter and activates *OsREM20* expression (Wu et al., 2021). However, whether *OsREM20* regulates rice inflorescence architecture through controlling rice meristem identity conversion and how OsMADS34 activates *OsREM20* expression as a transcriptional repressor is unknown.

*TAWAWA1* (*TAW1*) encodes a protein with an unknown function that influences rice inflorescence architecture by blocking the transition from BM to SM (Yoshida et al., 2013). *TAW1* is highly expressed in SAM and BM but disappears after BM formation. In the gain-of-function mutant *tawawa1*-*d*, the IM activity is prolonged and spikelet formation is delayed due to the continuously high expression of *TAW1* in BM and SM, resulting in branch extension and spikelet number increase. On the contrary, reduced *TAW1* expression causes IM prematuration and early formation of spikelets, producing small inflorescence (Yoshida et al., 2013). *TAW1* acts upstream of the *SVP* genes and positively regulates their expression to suppress SM identity (Yoshida et al., 2013). GROWTH-REGULATING FACTOR6 (OsGRF6) directly binds the promoter of *TAW1* and *OsMADS34*, positively controlling secondary branch number. Overexpression of *OsGRF6* leads to the increase of both primary and secondary branches, while knocking down *OsGRF6* generates abnormal inflorescences with no secondary branch (Gao et al., 2015). *miR396b* targets *OsGRF6* and suppresses its expression. Consistent with the role of *OsGRF6*, plants overexpressing the target mimicry of *miR396b* generate more secondary branches, but the *miR396b* overexpression plants have no secondary branch. Both *TAW1* and *OsMADS34* are up-regulated in the *miR396b* mimicry plants, suggesting that OsGRF6 could activate those two genes (Gao et al., 2015). Because *TAW1* and *OsMADS34* play opposing roles in determining rice inflorescence architecture, the function of *OsGRF6* may be mediated predominantly by *TAW1*, at the very least by balancing *TAW1* and *OsMADS34* expression.

## Control of branch elongation

Panicle branch elongation is another crucial event during rice inflorescence development. Although the development of BM has been extensively studied, the research on branch elongation in rice is still quite limited.

*SHORT PANICLE1* (*SP1*)/*PANICLE LENGTH3* (*PAL3*) encodes a polypeptide transporter located on the plasma membrane and is involved in the regulation of rice panicle branch elongation. Development of IM is normal in the loss-of-function mutants *sp1*/*pal3*, but in the later stage, the branches of the *sp1*/*pal3* mutants could not extend properly, resulting in delayed elongation or even degeneration of branches and subsequently short inflorescences (Li et al., 2009; Shang et al., 2021). *SP1* is highly expressed in the branch phloem of the inflorescence, consistent with its role in branch elongation (Li et al., 2009).

*DEP2* (DENSE AND ERECT PANICLE2) influences the elongation of the main axis and inflorescence branches. The *dep2* mutant generates the dense and erect inflorescence. *DEP2* encodes a plant-specific protein with unknown function and is highly expressed in rachis and branches of the young panicle. Several cell cycle genes are down-regulated in the *dep2* mutant, suggesting that cell proliferation is affected (Li et al., 2010).

Gibberellins (GAs) play a vital role in controlling organ elongation including rice inflorescence. This topic will be discussed in the ‘Gibberellins’ section below.

## Roles of phytohormones in controlling inflorescence architecture

Phytohormones are tiny regulatory molecules that affect almost all aspects of plant growth and development. Rice inflorescence formation and development are regulated by a range of hormones, such as cytokinins, auxin and gibberellins. These hormones frequently communicate with and affect one another, making them essential components of rice yield attributes.

### Cytokinins

**Figure 4:**
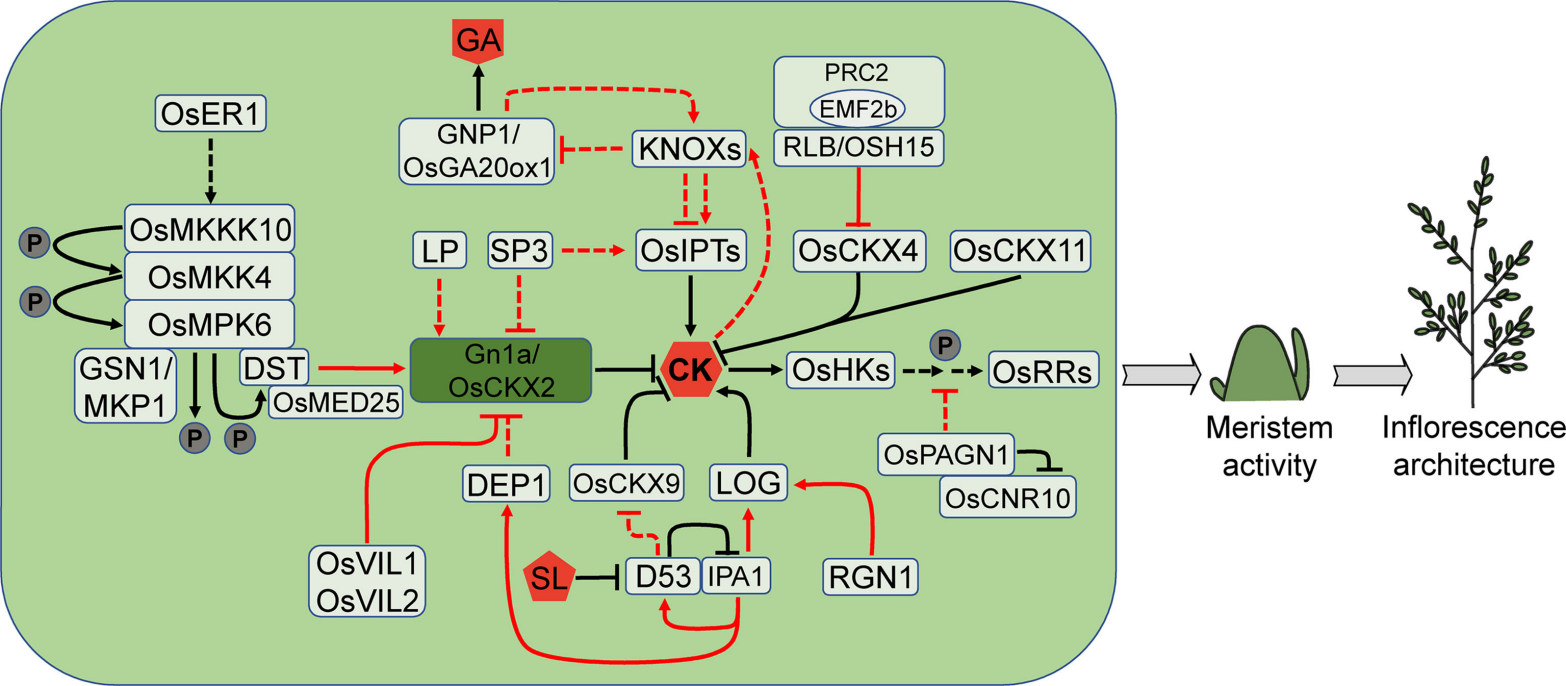
CK pathways that are involved in rice meristem activity. CK stimulates meristem activity and has the potential to increase yield. The CK levels are modulated by several enzymes, such as OsCKXs, LOGs and OsIPTs. Noticeably, *Gn1a/OsCKX2*, as a master regulator of rice inflorescence size and yield, is controlled by numerous other factors. CK also collaborates with other hormones such as GA and SL, in controlling rice inflorescence architecture. Arrows and blocked arrows represent positive and negative regulation, respectively. Solid and dashed lines represent direct and indirect regulation, respectively. Red and black lines represent regulation between factors at transcriptional and protein level, respectively. P, phosphorylation; CK, cytokinin; GA, gibberellin; SL, strigolactone.

Cytokinins (CKs) are adenine-derived compounds that are mainly involved in plant cell division (Werner et al., 2001; Schaller et al., 2014; Yang et al., 2021), playing a crucial role in many plant development processes, such as promoting the initiation and maintenance of SAM, regulating the development of flower organs, and determining the root meristem size (Wybouw and De Rybel, 2019). Many genes have recently been discovered to regulate CK homeostasis and signaling, which affect meristem activity and inflorescence development in rice (**Figure 4**).

The CK signaling transduction is mediated by the two-component system (TCS) that includes receptor histidine kinases (HKs), histidine phosphotransfer proteins (HPts) and response regulators (RRs). Knockout of rice CK receptors OsHK5/OHK3 or OsHK6/OHK5 impairs various aspects of rice development, including root and shoot growth (Burr et al., 2020). The panicle length, branch number and spikelet number are all reduced in the *hk5* and *hk6* single mutants, and the *hk5 hk6* double mutant displays more severe defects. These abnormalities in the *hk* mutants probably arise from the poor establishment of IM (Burr et al., 2020). Type-B RRs, as transcription factors, function downstream of HKs (Sakai et al., 2001; Mason et al., 2005; Zubo et al., 2017; Xie et al., 2018). The rice *rr21 rr22 rr23* triple mutant produces shorter inflorescences and fewer branches, leading to the reduction in spikelet number (Worthen et al., 2019). Type-A RRs are primary responsors to CK signaling and regulated by type-B RRs (Brandstatter and Kieber, 1998; Rashotte et al., 2003; Brenner et al., 2005). Overexpression of type-A *OsRR6* generates small inflorescences with fewer branches and spikelets (Hirose et al., 2007).

*PLANT ARCHITECTURE AND GRAIN NUMBER1* (*OsPAGN1*) encodes a RING U-box protein, likely an E3 ubiquitin ligase. Knockout of *OsPAGN1* results in an increase in primary branch number and grain number. The expression of several CK signaling genes including type-A *OsRR9*/*10*, HPt gene *OsAHP1* and *OsAHP2* is elevated in the *pagn1* mutant, implying that *OsPAGN1* may negatively regulate CK signaling. OsPAGN1 interacts with CELL NUMBER REGULATOR10 (OsCNR10) and probably ubiquitinates OsCNR10 for degradation. However, it is very perplexing that the *cnr10* mutant shows similar changes to the *pagn1* mutant in inflorescence architecture (Yan et al., 2022). Other regulators may be involved in the interaction of the two proteins.

*GRAIN NUMBER 1a* (*Gn1a*), one major QTL controlling rice grain number per panicle, encodes CK oxidase/dehydrogenase OsCKX2 which can irreversibly degrade the active CK into adenine or adenosine and side chain. *Gn1a* is predominantly expressed in the vascular tissue of young panicles and culms. The decreased *Gn1a* expression leads to the accumulation of CKs in IM and the increased meristem activity, resulting in the increased grain number and yield (Ashikari et al., 2005).

Numerous genes so far have been discovered to influence CK contents in IM through modulating *Gn1a* expression, which consequently affects inflorescence architecture and yield. *DEP1* is another major QTL that controls rice inflorescence architecture. The dominant allele *dep1* leads to truncation of the phosphatidylethanolamine-binding protein-like domain protein, resulting in increased meristem activity, short and erect inflorescence. In the NIL-*dep1* plants, *Gn1a* expression is decreased significantly, indicating that *DEP1* may affect meristem activity by regulating CKs accumulation (Huang et al., 2009). *SP3* can affect CK contents in young inflorescence. The expression of CK synthesis gene ISOPENTENYL TRANSFERASEs (OsIPTs) is decreased in the *sp3* mutant, but the degradation genes, including *OsCKX2* and other *OsCKXs*, are up-regulated, resulting in a drop in trans-zeatin (tZ) and a reduction in inflorescence size (Huang et al., 2019). *LARGER PANICLE* (*LP*) encodes a kelch repeat-containing F-box protein that negatively regulates inflorescence size by affecting *OsCKX2* expression. The loss of function of *LP* leads to an increase in branch number and grain number, which is most likely attributed to up-regulation of CK levels (Li et al., 2011). The chromatin interacting factor VIN3-LIKE1 (OsVIL1) and OsVIL2 directly binds to the promoter of *OsCKX2* and regulates H3K27 methylation, resulting in decreased *OsCKX2* expression, increased CK levels and an increase in the number of branches and grains (Yang et al., 2019; Yoon et al., 2021).

*OsCKX2* promoter can also be bound by the zinc finger transcription factor DROUGHT AND SALT TOLERANCE (DST). *REGULATOR OF Gn1a* (*REG*), the semi-dominant allele of *DST*, disrupts *OsCKX2* expression mediated by *DST*, leading to higher CK levels in the IM, increased meristem activity, and more branches and grains (Li et al., 2013). DST physically interacts with the Mediator complex subunit 25 (OsMED25) which is probably a coactivator of DST. The inflorescence phenotype of *OsMED25* knock-down plants and the *osmed25* mutant is similar to the *dst*/*reg* mutant. The DST-OsMED25 complex recruits RNA polymerase II (Pol II) to activate *OsCKX2* expression (Lin et al., 2022). The DST-OsCKX2 module for inflorescence development is also regulated by the OsMKKK10-OsMKK4-OsMPK6 cascade signal which adversely affects spikelet formation (Guo et al., 2018; Guo et al., 2020). OsMPK6 interacts with and phosphorylates DST, enhancing the transcriptional activation capacity of DST on *OsCKX2*, boosting CK degradation and maintaining normal CK levels during inflorescence development (Guo et al., 2020). The receptor-like kinase ERECTA1 (OsER1) acts upstream of the OsMKKK10-OsMKK4-OsMPK6 cascade signal and modulates OsMPK6 phosphorylation level. The *oser1* loss-of-function mutant has a lower level of OsMPK6 phosphorylation and produces more spikelets. OsMPK6 interacts with and is dephosphorylated by the mitogen-activated protein kinase phosphatase GRAIN SIZE AND NUMBER1 (GSN1)/OsMKP1, resulting in deactivation which positively regulates spikelet number (Guo et al., 2018). Suppressing *GSN1* expression causes decreased spikelet number, while overexpression leads to increased spikelet number. The *gsn1 osmpk6* double mutant produces much more spikelets than the *gsn1* mutant, but fewer than the *osmpk6* mutant (Guo et al., 2018; Guo et al., 2020).

Overexpression of *OsCKX4* also leads to changes in inflorescence architecture as demonstrated by sparser and smaller panicles (Wang et al., 2022). The KNOX protein RICE LATERAL BRANCH (RLB)/OSH15 epigenetically suppresses *OsCKX4* expression through recruiting OsEMF2b, a component of the polycomb repressive complex 2 (PRC2) which mediates H3K27 tri-methylation on target genes. The loss-of-function mutant *rlb* has shorter inflorescences with reduced spikelets, due to the decrease of secondary branches (Wang et al., 2022).

It is intriguing that either knockout or overexpression of another CK oxidase/dehydrogenase gene *OsCKX9* leads to the decrease of panicle length, number of branches and grains (Duan et al., 2019b). This reveals that the steady-state levels of the *OsCKX9* expression may play a key role in the regulation of rice inflorescence architecture. However, *OsCKX9* may not directly regulate inflorescence size but through regulating tillering. The smaller panicle is perhaps a tradeoff effect of increased tiller number. *OsCKX9* is a primary responsor to the strigolactone (SL) signaling as its expression is up-regulated within 1 h by SL treatment. This induction is dependent on the SL signaling repressor DWARF53 (D53) which inhibits the IPA1 transcriptional activation activity by interacting with it. In turn, IPA1 directly regulates *D53* expression, forming a negative feedback loop (Song et al., 2017; Duan et al., 2019b). In the shoot base of the gain-of-function *d53* mutant, the *OsCKX9* expression is decreased, leading to the significant increase of CK contents (Duan et al., 2019b).

Another CK oxidase/dehydrogenase gene *OsCKX11* simultaneously mediates leaf senescence and grain number. The *osckx11* mutant shows delayed leaf senescence under dark treatment and produces a larger inflorescence with more primary branches and high grain yield. Expression pattern analysis shows that *OsCKX11* is highly expressed in PBM, SBM and SM (Zhang et al., 2021).

CK synthesis involves two families of key genes, OsIPTs and *LONELY GUYs* (*LOGs*). *LOG* encodes CK-activating enzyme, which directly converts inactive CK nucleosides into free active CKs. *LOG* is expressed at a low level in a small region of upper SAM but highly in BM and SM. In the *log* mutant, the IM ceases soon after generating a modest amount of lateral meristem in the reproductive stage, resulting in a reduction of inflorescence size and fewer branches and spikelets (Kurakawa et al., 2007). The *LOG* expression is directly regulated by the R2R3 MYB transcription factor REGULATOR OF GRAIN NUMBER1 (RGN1). The mutation of *RGN1* leads to the absence of lateral spikelets on secondary branches (Li et al., 2022). The promoter of *LOG* is also bound by IPA1 (Lu et al., 2013; Du et al., 2017), implying that IPA1 mediates inflorescence architecture by altering CK levels directly or indirectly.

CKs may maintain SAM activity in rice through controlling the expression of *KNOXs*, which are induced by CKs (Tsuda et al., 2011). Overexpression of CK signalling genes, such as *OHK3*, *OHP2* and type-B *RRs*, promotes CK-mediated induction of *OSH1*. However, the response of *OSH1* to exogenous CKs lags behind that of the CK primary response genes, type-A *RRs*, suggesting that type-B RRs may not directly regulate *OSH1* (Naruse et al., 2018). In the formation and maintenance of rice SAM, there appears to be a complicated connection between the CK synthesis genes *OsIPTs* and *KNOXs*. Increasing *KNOXs* expression induces *OsIPT2* and *OsIPT3*, but inhibits other *OsIPTs*. In the aboveground tissues, overexpression of *OSH1* and *OsIPT3* results in comparable phenotypes. Apart from upregulating the CK synthesis genes, *KNOXs* inhibit expression of the gibberellins (GAs) synthesis genes *OsGA20oxs*, permitting the meristem to maintain a state of high-level CKs and low-level GAs, which is required for meristem establishment and maintenance (Sakamoto et al., 2006).

### Auxin

**Figure 5:**
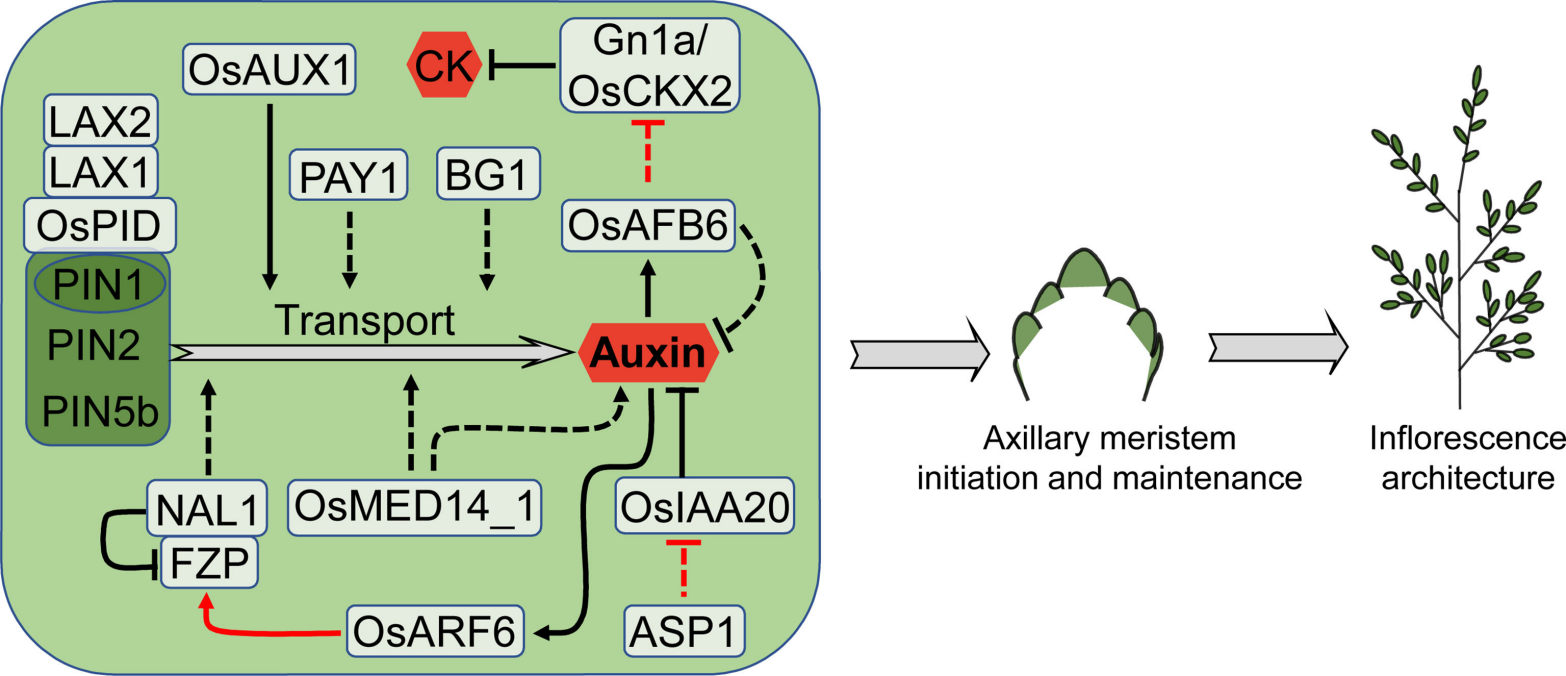
Factors involved in auxin-mediated regulation of rice inflorescence architecture. Auxin plays a vital role in axillary meristem initiation and maintenance at both vegetative and reproductive stage. PINs-mediated auxin transport is a crucial process for auxin function. Auxin also interacts with CK in determining rice inflorescence architecture. Arrows and blocked arrows represent positive and negative regulation, respectively. Solid and dashed lines represent direct and indirect regulation, respectively. Red and black lines represent regulation between factors at transcriptional and protein level, respectively. CK, cytokinin.

Auxin is the first plant hormone identified, and it is involved in many aspects of plant growth and development, such as embryogenesis, root structure, geotropism, phototropism, and the formation of plant lateral organs (Rashotte et al., 2000; Reinhardt et al., 2000; Benková et al., 2003; Blakeslee et al., 2004; Möller and Weijers, 2009). Auxin is mainly synthesized in the shoot tip and transported downward by polar transport, indirectly suppressing the formation of axillary buds (Sieberer and Leyser, 2006; Zwiewka et al., 2019). During the formation of the rice inflorescence, auxin is involved in the initiation and maintenance of the axillary meristem, which influences the development of panicle branches (Deveshwar et al., 2020) (**Figure 5**).

Auxin transport through tissues is essential for many aspects of plant development. This movement is regulated by auxin transporters, among which the PIN-FORMED (PIN) protein family plays a vital role in accelerating the outward transport of auxin from cells (Zazímalová et al., 2010). PIN1 is the first auxin efflux carrier discovered in Arabidopsis, having polarity in the plasma membranes of root, stem, inflorescence axis, and embryo cells, and its loss-of-function mutant has a substantial impact on organ initiation (Okada et al., 1991; Gälweiler et al., 1998; Friml et al., 2003; Blilou et al., 2005; Adamowski and Friml et al., 2015). There are four PIN1 homologues (PIN1a-PIN1d) in rice. No evident phenotypic change is observed in any single *pin1* mutant, but the *pin1a pin1b* double mutation changes root architecture and results in a wider panicle branching angle; the *pin1c pin1d* double mutant has fewer branches and no spikelet (Li et al., 2019; Liu et al., 2022). Overexpression of *OsPIN2*, another member of *PIN* family, promotes auxin transport from the shoot to the root-shoot junction, causing a higher non-tissue-specific concentration of free auxin at the root-shoot junction. This non-specific auxin accumulation gives rise to reduced plant height, increased tillering, shorter panicles and fewer grains (Chen et al., 2012). Another member of PIN family OsPIN5b participates in auxin homeostasis, transportation and distribution, thereby regulating rice plant architecture and yield (Mravec et al., 2009; Barbez et al., 2012; Lu et al., 2015a). *OsPIN5b* overexpression causes diverse morphologies, including decreased plant height, fewer tillers, lower seed-setting rate and shorter panicles. On the contrary, knockdown of *OsPIN5b* produces more tillers, a better developed root system and longer panicles (Lu et al., 2015a).

Serine/threonine protein kinase OsPINOID (OsPID) interacts with PIN1a and PIN1b to govern polar transport and distribution of auxin, and modulates the formation and development of rice flower organs. *OsPID* expression is high in young panicles and its overexpression plants have more panicle branches and grains (Wu et al., 2020). The bHLH transcription factor LAX PANICLE1 (LAX1) also interacts with OsPID, probably controlling inflorescence architecture through affecting auxin polar transport (Wu et al., 2020), and this mechanism is conserved in maize (Gallavotti et al., 2004). The expression of *LAX1* is not detected in SAM but restricted to the boundary between the inflorescence rachis and the region of new meristem formation. With the elongation of new meristem, *LAX1* expression is gradually diminished. The *lax1* mutant lacks lateral spikelets in favor of sole terminal spikelets (Komatsu et al., 2001). LAX1 interacts with LAX2, and their double mutations enhance the phenotype of the *lax1* mutant, indicating that *LAX1* governs the inflorescence meristem initiation by controlling auxin signal transduction and transport, either independently or in collaboration with *LAX2*. Unlike *LAX1*, *LAX2* expression is observed in PBM, SBM and SM, covering the expression domain of *LAX1* (Tabuchi et al., 2011).

By high-throughput single-cell RNA sequencing technology, auxin influx transporter gene *OsAUX1* is identified to have enriched expression in BM. Consistent with its expression pattern, the loss-of-function mutant of *OsAUX1* produces smaller inflorescence with reduced branch and spikelet number (Zong et al., 2022).

A few other genes also affect inflorescence architecture through modulating auxin transport. *PLANT ARCHITECTURE AND YIELD1* (*PAY1*) encodes a nuclear-localized peptidase, controlling rice plant architecture by influencing the auxin polar transport and the level of endogenous indole 3-acetic acid (IAA). Overexpression of *PAY1* produces a significant increase in grain number and yield (Zhao et al., 2015). *BIG GRAIN1* (*BG1*) encodes a membrane-localized protein that, when overexpressed, can increase grain and inflorescence size as well as yield through altering auxin response and transport (Liu et al., 2015a). OsMED14_1 is a subunit of the Mediator complex that regulates diverse biological processes. In the *OsMED14*_1 knock-down plants, auxin level and *PINs* expression decreased, giving rise to fewer primary and secondary branches as well as spikelet (Malik et al., 2020). *NARROW LEAF1* (*NAL1*) encodes a trypsin-like serine/cysteine protease, which controls rice leaf width by regulating auxin polar transport activity (Qi et al., 2008; Jiang et al., 2015). Overexpression of the *NAL1* alleles *LSCHL4* and *SPIKELET NUMBER* (*SPIKE*) results in larger inflorescences and higher yields (Fujita et al., 2013; Zhang et al., 2014). NAL1 interacts with FZP and promotes the degradation of FZP. Down-regulation of *FZP* or up-regulation of *NAL1* increases the number of secondary branches, grains per panicle and yield per plant (Huang et al., 2018).

Auxin receptor TRANSPORT INHIBITOR RESPONSES (TIRs)/AUXIN SIGNALING F-box (AFBs) are a class of F-box protein. Overexpression of *OsAFB6* in rice causes a rise in primary branches and spikelets due to reduced IAA levels and *Gn1a* expression, resulting in an increase in CK contents and IM activity (He et al., 2018). *AUXIN RESPONSE FACTORs* (*ARFs*) are auxin signal response factors, of which OsARF6 can directly bind to the promoter of *FZP* and activate its expression. A 4-bp tandem repeat deletion near the binding element affects the capacity of OsARF6 to regulate *FZP*, improving secondary branch number and grain yield (Huang et al., 2018). Similar to *TPL* in Arabidopsis, the meristem fate regulator *ASP1* is also related to auxin signaling (Szemenyei et al., 2008). In the *asp1* mutant, expression of the auxin signaling negative regulatory gene *OsIAA20* is up-regulated, indicating that auxin signal is interrupted (Yoshida et al., 2012).

### Gibberellins

**Figure 6:**
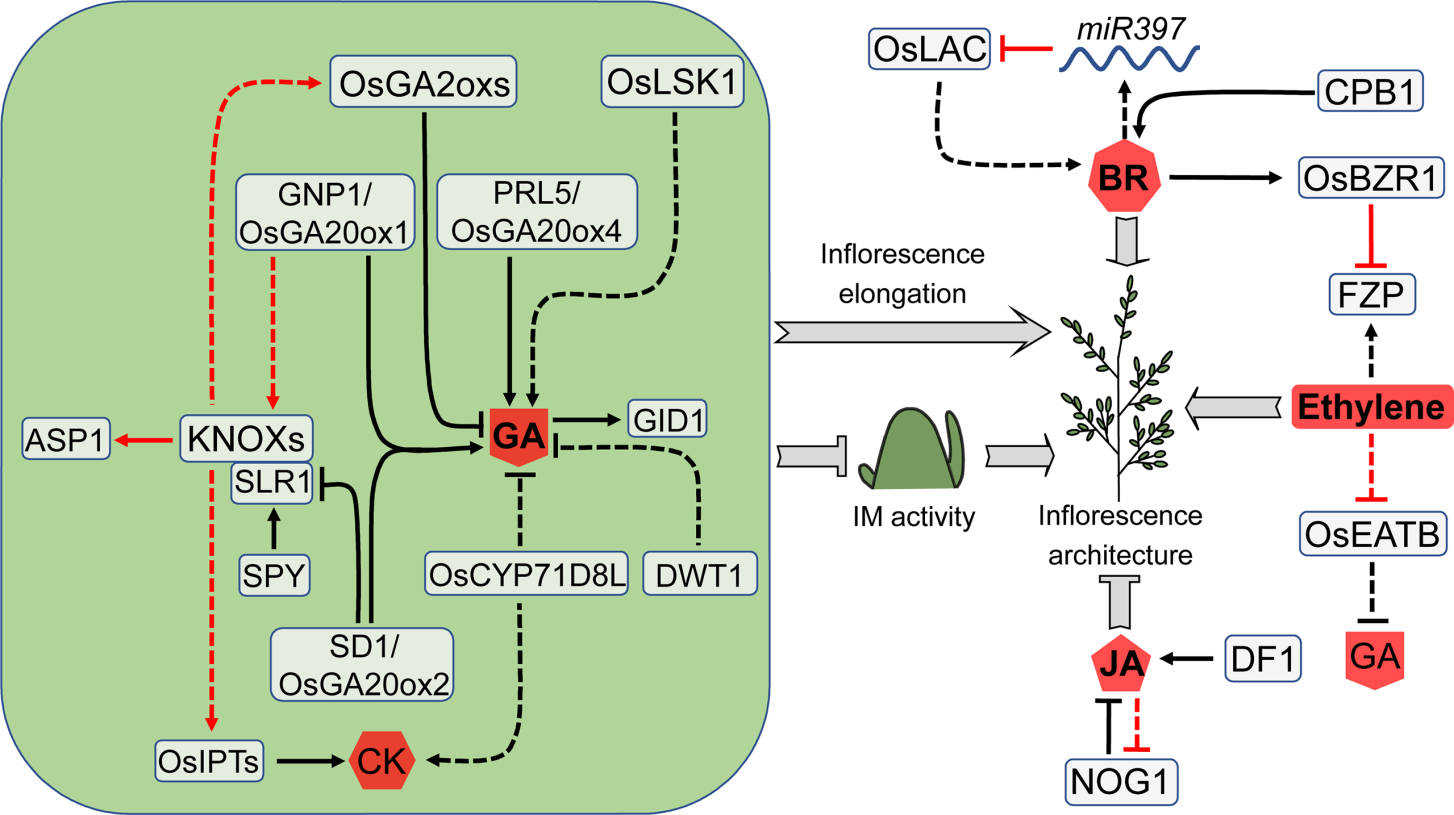
Factors involved in GA- and other hormone-mediated regulation of rice inflorescence architecture. Through an opposite way, GA promotes branch elongation or inhibits meristem activity to influence the rice inflorescences architecture. GA antagonizes CK in maintaining meristem activity, and *KNOX* genes act as coordinators between GA and CK. Several additional hormones also have a substantial impact on rice inflorescence architecture. These hormone regulators form a complicated network that influences one another. Arrows and blocked arrows represent positive and negative regulation, respectively. Solid and dashed lines represent direct and indirect regulation, respectively. Red and black lines represent regulation between factors at transcriptional and protein level, respectively. BR, brassinosteroid; CK, cytokinin; GA, gibberellin; JA, jasmonic acid.

Gibberellins (GAs) are a kind of tetracyclic diterpene hormones that regulate many aspects of plant development including organ elongation, seed germination and flowering (Cheng et al., 2004; Tyler et al., 2004; Kuroha et al., 2018). GA signaling is received by the receptor GIBBERELLIN INSENSITIVE DWARF1 (GID1) (Ueguchi-Tanaka et al., 2005). Loss-of-function mutations of *GID1* cause multiple phenotype changes including dwarfism, small inflorescence and reduced fertility (Ueguchi-Tanaka et al., 2005; Ayano et al., 2014; Wang et al., 2018). In recent years, several additional genes involved in GA synthesis and signaling have been discovered to influence rice inflorescence architecture by regulating IM activity or inflorescence branch elongation (**Figure 6**).

Studies in Arabidopsis and rice show that GAs antagonize CKs in maintaining SAM activity, and *KNOXs* act as coordinators between these two hormones (Hay et al., 2002; Jasinski et al., 2005; Sakamoto et al., 2006). *GRAIN NUMBER PER PANICLE1* (*GNP1*), a major QTL controlling the grain number in rice, encodes the GAs synthesis enzyme OsGA20ox1 and is expressed in SAM and BM (Wu et al., 2016b). *GNP1* upregulation in IM leads to an increase of *KNOXs* in a feedback manner, which activates *OsIPTs*. Subsequently, elevated CK levels and *KNOXs* trigger expression of the GA catabolic genes *GA2oxs*, leading to enhanced active GAs catabolism. Therefore, the unfavorable effect of GA production on meristem activity is decreased because GA_1_ and GA_3_ are not accumulated in IM. This equilibrium mechanism improves yield by enhancing IM activity through boosting CKs activity and decreasing GAs activity (Wu et al., 2016b).

Overexpression or gain-of-function mutation of the cytochrome P450 monooxygenase gene *OsCYP71D8L* leads to the reduction in panicle length and grain number (Zhou et al., 2020), probably due to decreased GA levels. Surprisingly, CK levels increase in *OsCYP71D8L* overexpression plants (Zhou et al., 2020), but these enhanced CK levels do not compensate panicle defects induced by decreased GA levels. This study indicates that *OsCYP71D8L* functions mainly through GA rather than CK in regulating inflorescence architecture.

The rice ‘Green Revolution’ gene *SEMI*-*DWARF 1* (*SD1*) encodes GA20ox2, which has been used in breeding for many years. Recent studies reveal that *SD1* also participates in inflorescence development. The loss of function of *SD1* causes a decrease in panicle length, branch number and grain number. Consistent with its function, *SD1* is expressed in elongated PBM, SBM, and SM. The level of DELLA protein SLR1, a negative regulator of GA signaling, is increased dramatically in the *sd1* mutant. SLR1 interacts with KNOXs to inhibit KNOXs-mediated activation of downstream genes, such as *ASP1* (Su et al., 2021). Although both SD1 and GNP1 belong to GA20 oxidases, they may work independently to regulate inflorescence architecture due to their diverse spatiotemporal expression patterns (Su et al., 2021). *PANICLE RACHIS LENGTH5* (*PRL5*) is a major QTL for rice panicle length, encoding another GA oxidases, OsGA20ox4. *PRL5* overexpression increases GA levels in the inflorescence, resulting in elongation of the panicle axis (Agata et al., 2020).

*SPINDLY* (*OsSPY*) encodes *N*-acetyl glucosamine transferase, a negative regulator of GA signaling by enhancing the SLR1 activity (Shimada et al., 2006; Olszewski et al., 2010). The R833L substitution of OsSPY at the conserved C-terminus of the enzymatic domain leads to the decreased *O*-fucosyltransferase activity of OsSPY to SRL1, consequently increased panicle length, primary and secondary branch number and spikelet number (Yano et al., 2019).

*LARGE SPIKE S-DOMAIN RECEPTOR LIKE KINASE 1 (OsLSK1)* is an s-domain receptor kinase. Overexpression of truncated *OsLSK1* increases the number of primary branches and grains, which is probably attributable to the upregulation of several critical genes involved in GAs synthesis and signaling (Zou et al., 2015). *DWARF TILLER1* (*DWT1*) encodes a WUS-like homeobox transcription factor. The *dwt1* mutant produces main culm with normal height and dwarf tillers with unelongated internodes. Aa a result, those two types of tillers generate large and small inflorescences, respectively. It seems that *DWT1* is a direct regulator of tiller growth with indirect effect on panicle size. Interestingly, the expression of *DWT1* is detectable in PBM and SBM but not in the elongating internode. The expression of GA20 oxidase genes is up-regulated in the *dwt1* mutant, and their responsiveness to GAs is weakened, suggesting that *DWT1* is directly or indirectly related to GA signaling pathway (Wang et al., 2014).

### Other hormones

In addition to the three vital hormones mentioned above, several additional hormones are also involved in the regulation of rice inflorescence architecture (**Figure 6**).

Ethylene is a gaseous hormone that regulates a variety of plant growth processes. In rice, ethylene influences several essential agronomic traits, including flowering, grain size, and grain filling (Yin et al., 2017). Ethylene response factors (ERFs) are transcription factors that regulate ethylene signal transduction and response, among which OsEATB mediates the crosstalk between GA and ethylene. *OsEATB* expression is inhibited by ethylene, and its overexpression reduces GA contents, shortens the panicle length but increases grain number per panicle. *OsEATB* inhibits the ethylene-induced GA response by downregulating GA synthase, ent-kaurene synthase A (Qi et al., 2011). FZP is also an ERF domain-containing protein. As described above, *FZP* mediates the transformation of meristem identity, which affects inflorescence architecture and yield (Komatsu et al., 2003).

Brassinosteroids (BRs) are steroid hormones that regulate a wide range of biological processes, including plant development and stress response (Yang et al., 2011; Zhao et al., 2013; Gao et al., 2014; Saini et al., 2015; Divi and Krishna, 2009). *DWARF11* (*D11*) encodes a cytochrome P450 protein that is involved in the BR biosynthesis. *CLUSTERED PRIMARY BRANCH 1* (*CPB1*) is a *D11* allele that controls the clustering of rice inflorescence branches and is highly expressed in young panicles. The *cpb1* mutant has clustered primary branches and elongated internodes at the base of the main axis (Wu et al., 2016a). *OsBZR1* is a primary regulator of BR signaling in rice, and its overexpression results in phenotypic alterations in anther and grain size, as well as an increase in grain number per panicle (Zhu et al., 2015). OsBZR1 inhibits *FZP* expression through binding to the CGTG motif which is located in an 18-bp fragment inserted 5.3 kb upstream of *FZP*, resulting in the increased number of branches and grains (Bai et al., 2017).

*OsLAC* encodes a laccase protein that modulates plant response to BRs. This protein may influence multiple rice development processes by controlling BR signals. The panicle length, branch number, and grain number are all decreased in the *OsLAC* overexpression plants (Zhang et al., 2013). *OsLAC* is a target of *miR397*. In contrast to *OsLAC* overexpression, *miR397* overexpression increases the number of branches and grains. Plants with high levels of *miR397* are more sensitive to BR treatment. Additionally, BR levels declined somewhat in *miR397* overexpression plants but increased dramatically in *OsLAC* overexpression plants (Zhang et al., 2013).

Jasmonic acids (JAs) and derivatives are lipid-derived hormones that control plant defense responses and development processes, such as seed germination, root growth, tuber formation, tendril curling, trichome initiation, reproduction and aging (Kessler et al., 2004; Browse and Howe, 2008; Browse, 2009; Acosta and Farmer, 2010; Wasternack and Hause, 2013). In rice, JA suppresses spikelet growth and reduces yield through adversely regulating spikelet development (Kim et al., 2009; Cai et al., 2014). *DOUBLE FLORET1* (*DF1*) is an allele of *EXTRA GLUME1* (*EG1*) which controls spikelet development, encoding a plastid lipase involved in JA biosynthesis (Cai et al., 2014; Ren et al., 2018). The *df1* mutant develops two complete florets with normal grain in one pair of glumes (Ren et al., 2018). *NUMBER OF GRAINS 1* (*NOG1*) encodes an enoyl-CoA hydratase/isomerase that is involved in the regulation of JA synthesis and β-oxidation of fatty acid. A 12-bp insertion in the *NOG1* promoter enhances *NOG1* expression, resulting in higher levels of enoyl-CoA hydratase/isomerase, lower total fatty acid and linolenic acid levels, as well as lower JA levels. Changes in these components lead to more grains per panicle and higher yield. Excessively applying JAs reduces *NOG1* expression and yield, suggesting that JAs have a negative impact on *NOG1* expression and rice yield (Huo et al., 2017).

## Conclusions and perspectives

Owing to the importance of rice inflorescence architecture for grain yield, studies on inflorescence development have risen remarkedly in recent years, and multiple relevant genes have been identified. These genes mainly function in meristem activity maintenance, meristem identity conversion, and phytohormones regulation. Nevertheless, several other crucial genes involved in rice inflorescence architecture, such as *OsPDCD5* (Dong et al., 2021), *OsTPR* (Pasion et al., 2021), *OsKNR2* (Chen et al., 2022) etc. are not discussed here due to their unclear or unrelated genetic mechanism. Although so many genes related to rice inflorescence development have been identified, how can the revealed genetic pathways be fine-tuned to create an ideal inflorescence architecture to maximize the yield? It is not easy to answer this question.

The first challenge is environment cues, such as light, temperature, humidity and nutrients. The ideal inflorescence architecture should be appropriate for local ecological niche. For example, the inflorescence of high-yielding *japonica* varieties grown in northern China is generally dense and erect, due to the gain-of-function mutation of *DEP1* (Huang et al., 2009). Despite of the potential of improving grain yield, this allele is rarely used in *indica* varieties grown in southern China (Huang et al., 2009). One of the possible reasons could be that dense and erect panicle is prone to diseases, such as false smut, under the high temperature and humidity in southern China (Sun et al., 2020). On the contrary, the *indica* rice generates long and drooping inflorescences that provide more space for spikelets, establishing a microenvironment to prevent pathogen attacks.

Meanwhile, the tradeoff between the inflorescence architecture and other traits severely restricts breeding. In many cases, this tradeoff is controlled by common genes. *FUWA* encodes a protein that contains an NHL domain. The mutation of *FUWA* causes short and erect panicles, while the grains become smaller and thicker (Chen et al., 2015). *DEP1*, *DEP2* and *DEP3* also influence grain size apart from inflorescence architecture (Huang et al., 2009; Li et al., 2010; Qiao et al., 2011). Knockout of *OsSPL4* or overexpression of *OsSPL13* enlarges both inflorescences and grains (Si et al., 2016; Hu et al., 2021b). The loss of function of *MOC1*, *LAX1*, and *LAX2* simultaneously reduces the number of tillers and panicle branches (Komatsu et al., 2001; Li et al., 2003; Tabuchi et al., 2011). Because the impacts of these genes on inflorescence architecture and other traits are not always beneficial to yield, resolving the tradeoff between inflorescence size and other morphologies to enhance yield is an issue worth investigating. Recently, a study addressed tradeoff effects between inflorescence size and tiller number. Using tilling-deletion-based screen for the *IPA1* promoter by CRISPR-Cas9, a 54-bp deletion that contains an AN-1 binding site in the *IPA1* promoter simultaneously increases tiller number and inflorescence size (Song et al., 2022). This research reveals a possibility for breeders that the association between multiple traits can be dissected, and genetic effects can be precisely modified.

Phytohormone is another challenge for creating ideal inflorescence architecture, owing to its intricacy and cross-talk at various regulatory levels. Auxin promotes axillary meristem initiation, and its efflux determines both inflorescence branch number and branch angle (Li et al., 2019; Liu et al., 2022). CK positively regulates inflorescence meristem activity, while GA is detrimental for meristem activity. This antagonism is linked by *KNOXs* and *GNP1* (Jasinski et al., 2005; Sakamoto et al., 2006; Wu et al., 2016b). Apart from inflorescence architecture, ethylene, brassinosteroids, and JA usually act on other biological processes, such as grain filling, leaf angle and stress response (Yin et al, 2017; Tong and Chu, 2018). Pyramiding the pleiotropic positive effects of hormones by optimizing their levels or balancing their connections will be a challenging task in the future.

## Author contributions

YC and AK wrote the manuscript. XL made valuable suggestions and revised the manuscript. All authors read and approved the final manuscript.

## Funding

Research in our lab was funded by the Agricultural Variety Improvement Project of Shandong Province, China (grant number 2021LZGC020), and the Agricultural Science and Technology Innovation Program of Chinese Academy of Agricultural Sciences.

## Acknowledgments

We thank China Scholarship Council (CSC) for providing full scholarship to AK for his Master and Ph.D. study, respectively.

## Conflict of interest

The authors declare that the research was conducted in the absence of any commercial or financial relationships that could be construed as a potential conflict of interest.

## Publisher’s note

All claims expressed in this article are solely those of the authors and do not necessarily represent those of their affiliated organizations, or those of the publisher, the editors and the reviewers. Any product that may be evaluated in this article, or claim that may be made by its manufacturer, is not guaranteed or endorsed by the publisher.
